# Examining a Sequential Mediation Model of Chinese University Students’ Well-Being: A Career Construction Perspective

**DOI:** 10.3389/fpsyg.2018.00593

**Published:** 2018-04-25

**Authors:** Mingke Zhuang, Zhuolin She, Zijun Cai, Zheng Huang, Qian Xiang, Ping Wang, Fei Zhu

**Affiliations:** ^1^School of Psychological and Cognitive Sciences, Peking University, Beijing, China; ^2^School of Economics and Management, Tsinghua University, Beijing, China; ^3^Business School, Faculty of Arts, Business, Law and Education, University of Western Australia, Perth, WA, Australia; ^4^Key Laboratory of Mental Health, Institute of Psychology, Chinese Academy of Sciences, Beijing, China; ^5^School of Environment and Natural Resources, Renmin University of China, Beijing, China; ^6^School of Government, Peking University, Beijing, China; ^7^Business School, Central University of Finance and Economics, Beijing, China

**Keywords:** career construction theory, career adaptability, meaning in life, well-being, emotions

## Abstract

Despite career construction theory attends to individual subjective career and provides a useful lens to study well-being, extant research has yielded limited insights into the mechanisms through which career construction variables influence individual well-being. To address this important gap, the present study examined a mediation model that links indicators of career adaptivity (big-five personality and approach/avoidance traits) to psychological well-being (psychological flourishing and life satisfaction) through career adaptability and in sequent meaning of life (presence of life meaning and search for life meaning) among a sample of Chinese university students (*N* = 165). The results of a two-wave survey study showed that career adaptability and presence of life meaning mediated the effects of openness to experience, consciousness, approach trait, and avoidance trait on individual well-being in sequence. The results also showed that approach trait’s effect on presence of meaning was partially mediated by career adaptability; career adaptability’s effect on psychological flourishing was partially mediated by presence of meaning. These findings advance understanding of antecedents to individual well-being from a career construction perspective, and carry implications for career education and counseling practices.

## Introduction

It has been widely accepted that career experience plays an important role in promoting and sustaining psychological well-being (e.g., Savickas, 2005, 2013; Duffy et al., 2016), which refers to the experienced happiness and satisfaction in one’s life (Diener et al., 1985). University students are at the career exploration stage with a lot of uncertainties about their future career development, which have significant impact on their psychological well-being (Arnett, 2000; Savickas, 2002). From a career construction perspective (Savickas, 2005, 2013), to deal with the uncertainties at this stage, individuals need to possess relevant psychological capabilities to adapt to the vocational challenges (Savickas, 1997; Savickas et al., 2009). Career adaptability, the self-regulation resources that help individuals achieve adaptive outcomes such as career success (Zacher, 2014;

Guan et al., 2015c), reflect such capabilities. The role of career adaptability in predicting well-being indicators, such as life satisfaction, has been revealed in recent research (e.g., Buyukgoze-Kavas et al., 2015; Konstam et al., 2015). As such, scholars proposed that a deeper understanding of how career adaptability affects individual well-being can “equip career practitioners with new and refined tools for serving a wider range of students and clients” (Brown and Lent, 2016, p. 559).

In detail, career construction theory (Savickas, 2013) proposes that individuals use their vocational resources, including career adaptivity (i.e., individuals’ personalities that reflect their willingness and motivation to change) and career adaptability (i.e., individuals’ self-regulation resources to deal with vocational challenges and difficulties), to adapt to the environments. Successful adaptation, in turn, should positively predict individuals’ career performance and satisfaction. Accordingly, career adaptability is posited to mediate the effects of career adaptivity on individual well-being. Although previous research has identified that career adaptability serves as an important predictor for indicators of well-being, such as life satisfaction (see meta-analysis by Rudolph et al., 2017), not much work has been done to test this mediation model. Besides, previous studies have shown that meaning of life is a core psychological mechanism to promote well-being (Morgan and Farsides, 2009). We thus investigate whether students with high career adaptability are more likely to feel meaningful in life and achieve a high level of well-being. Although previous research has provided ample evidence for the contention that career adaptability positively predicts well-being, it is still largely unknown why this is the case (Hartung and Taber, 2008).

Last, well-being is a broad concept and can be operationalized in different ways; however, extant research mainly focused on the specific dimensions (e.g., Hirschi, 2009; Maggiori et al., 2013), rather than the integrative indicators of well-being, such as psychological flourishing (Diener et al., 2010), which refers to a state of optimal mental health that extends beyond merely the absence of mental illness (Keyes, 2007). This represents a significant gap because psychological flourishing captures both subjective (e.g., life satisfaction) and psychological components (e.g., personal growth) of well-being (Huppert and So, 2013), making it better reflect one’s level of well-being than any dimensional indicators. Thus, taking this integrative indicator into consideration could better and more accurately examine the effects of career adaptability on well-being.

**FIGURE 1:**
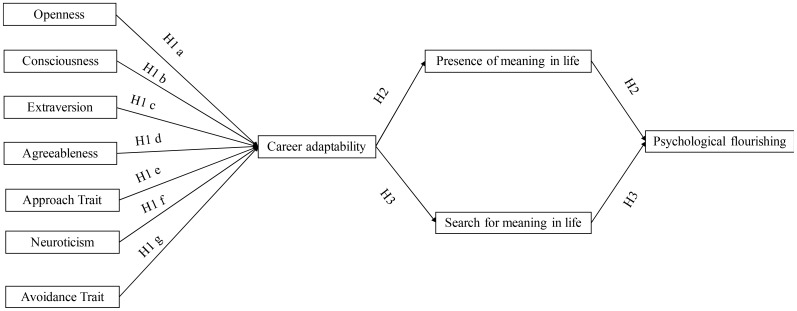
The proposed sequential mediation model.

A two-wave survey study was conducted among Chinese university students to test the sequential mediation model that links indicators of career adaptivity (big-five personality and approach/avoidance traits), career adaptability, meaning of life (presence of life meaning and search for life meaning), and individual well-being (psychological flourishing). Due to the booming economy and rapid social changes in the Chinese context, the new generation of Chinese university students are facing various opportunities and challenges in their career development. In addition, due to the prevalence of collectivistic values in China, Chinese university students’ career development is also influenced by social contexts, such as parental behaviors (e.g., Hofstede, 2001; Guan et al., 2015a,b). These structural and social contexts may result in high levels of stress among Chinese students. A recent survey study suggested that the percentage of Chinese university students who suffer from mental problems was from 5 to 7% (Sun, 2012). Under this background, this study thus has important implications for promoting Chinese students’ well-being. The proposed model is shown in **Figure 1**.

### Career Construction Theory

Career construction theory provides an integrative model to comprehend individuals’ vocational behaviors (Savickas, 2005, 2013). It asserts that continuous adaptation to the work environment is crucial for career performance and satisfaction. Specifically, individuals who are willing (career adaptivity) and able (career adaptability) to perform behaviors (adapting response) will show higher levels of adaptation (outcomes) (Savickas and Porfeli, 2012). Career adaptivity refers to the willingness/flexibility to adapt through making changes. Career adaptability is considered as individual strengths to handle difficulties and challenges during career development. Adapting response entails adaptive behaviors to address changing conditions and manage new career situations. Adaptation results capture the goodness of person–environment fit and can be indicated by career development, success and satisfaction (Savickas and Porfeli, 2012).

Accordingly, career construction theory suggests that individuals’ adaptivity positively impacts their career adaptability which, in turn, positively relates to adapting responses and adaptation results. In the present study, basic traits (i.e., big-five personality, the approach/avoidance traits) serve as important indicators of adaptivity (Zacher, 2014; Guan et al., 2017a), while meaning in life and individual well-being represent the results of adaptation. Therefore, we propose that basic traits predict individual career adaptability, which in turn relate to meaning in life and individual well-being.

### Basic Traits, Career Adaptability, and Meaning in Life

Career adaptability represents individuals’ psychological resources (i.e., concern, control, curiosity, and confidence) that enable them to cope with anticipated and current vocational challenges, transitions, and trauma (Savickas and Porfeli, 2012). Prior studies have revealed that this construct can be applied to different cultural contexts (Savickas and Porfeli, 2012). In the Chinese context, career adaptability has been found to be related to career-related skills (Guan et al., 2013, 2014), career satisfaction (Xie et al., 2016), and career success (Guan et al., 2015c).

In career construction theory, career adaptivity is “the personality trait of flexibility or willingness to change” (Savickas and Porfeli, 2012, p. 662), which serves as an important antecedent of career adaptability. It has been suggested that career adaptivity can be reflected by various dispositional factors (Savickas and Porfeli, 2012). In the present study, we specifically focus on basic traits such as the big-five personalities (McCrae and Costa, 1987) and approach/avoidance traits (Elliot and Thrash, 2002) as the indicator of career adaptivity, because previous studies (e.g., Savickas, 2013; Nilforooshan and Salimi, 2016) showed that they can well serve as the indicators of adaptivity. Especially, a systematic investigation of the trait basis of career adaptability by Guan et al. (2017a) has found that big-five personality and approach/avoidance traits serve as fundamental traits that reflect individuals’ adaptivity.

Among the five factors of personality, it has been found that career adaptability was positively associated with conscientiousness, extraversion, agreeableness, and openness to experience, but negatively associated with neuroticism (Teixeira et al., 2012; Zacher, 2014; Li et al., 2015; Guan et al., 2017a). The approach/avoidance trait model offers a motivational perspective to personality theories and complements the big-five personality model in explaining individual differences (Elliot and Thrash, 2002). People with approach trait tend to be sensitive to current or imagined positive stimuli while those with avoidance trait will be sensitive to current or imagined negative stimuli. As such, approach trait may sustain individuals’ abilities to deal with career challenges and increase their career adaptability, whereas avoidance trait may inhibit individuals’ abilities to solve career difficulties or barriers and decrease their career adaptability. Previous studies have offered supportive evidence on the significant relations between approach/avoidance traits and career adaptability (Li et al., 2015; Guan et al., 2017a).

Meaning in life generally reflects one’s sense of the existence and it has been operationalized with two dimensions: presence of meaning in life and search for meaning in life (Steger et al., 2006). The former reflects a status when individuals clearly identify the connection between themselves and the world, as well as the goals and values of their lives (Steger et al., 2008). The latter reflects a status when individuals have strong desire and take efforts to search for and improve their understanding about the purpose of their lives (Steger et al., 2006). Steger et al. (2006) proposed that these two dimensions are independent to each other because each is not necessary or sufficient to the existence of the other. Consistently, the empirical studies by Steger et al. (2008) showed that while experiencing meaningfulness (presence), individuals still could have the desire to explore more by challenging themselves (search for). Thus, in the current study, we treat them as separate factors, rather than two ends of the same continuum.

Career construction theory posits that individuals construct their careers by imposing meaning on their vocational experiences (Savickas, 2005, 2013). Since career adaptability reflects one’s self-regulation resources in relation to career-related tasks (Savickas, 1997), people with a high level of career adaptability would purposely construct their experiences and refresh their understanding about themselves, in order to identify meaning from their career experiences. For example, Praskova et al. (2014, p. 127) argued that career adaptability enables the youth to “make decisions about their future career and life, realize their abilities, and formulate and pursue goals linked to reaching meaningful work as adult.” Thus, we propose that individuals with a higher level of career adaptability can effectively achieve a higher level of presence of life meaning (Buyukgoze-Kavas et al., 2015; Yuen and Yau, 2015).

On the other hand, we argue that career adaptability will also positively predict search for life meaning. Since career development is a life-span process and individuals have to face various career transitions at different developmental stages (Savickas et al., 2009; Savickas and Porfeli, 2012), they need to constantly refresh and search new meaning in their career development (Steger et al., 2008; Miller and Rottinghaus, 2014). Previous research has shown that individuals with a higher level of career adaptability tend to pursue challenging career goals (Yang et al., 2015), put more effort in developing their professional skills (Guo et al., 2014), and have more exploration activities (Li et al., 2015; Guan et al., 2017b). Taken together, it is likely that career adaptability facilitates individuals’ search for life meaning, and mediates the effects of adaptivity indicators on the search for meaning (Savickas, 2013). We thus propose the following mediation hypotheses:

*Hypothesis 1:* Career adaptability mediates the positive relationship between openness to experience (H1a), conscientiousness (H1b), extraversion (H1c), agreeableness (H1d), the approach trait (H1e) and meaning in life, and also mediates the negative relationship between neuroticism (H1f), the avoidance trait (H1g), and meaning in life.

### Basic Traits, Career Adaptability, Meaning in Life, and Well-Being

As some research documented, psychological flourishing is about one’s self-perceived success in the important areas of their social lives and also reflects one’s psychosocial status (Diener et al., 2010). Previous studies show that meaning in life is a core psychological mechanism that helps individuals derive their well-being (e.g., Bonebright et al., 2000; Morgan and Farsides, 2009). For example, Frankl (1963) suggested that presence of meaning in life facilitates individuals to experience self-esteem, self-fulfillment and positive self-hood, and decrease their negative effects of apathy, boredom, and aimlessness (Morgan and Farsides, 2009), thus contributes to their psychological flourishing. Taken together, we propose that:

*Hypothesis 2:* Career adaptability and presence of meaning in life sequentially mediate the relationships between openness to experience (H2a), conscientiousness (H2b), extraversion (H2c), agreeableness (H2d), the approach trait (H2e), neuroticism (H2f), the avoidance trait (H2g), and psychological flourishing (i.e., individuals’ basic traits → career adaptability → presence of meaning in life → psychological flourishing).

In contrast, search for life meaning was found to have mixed effects on well-being. Though conceptually some researchers argued that search for life meaning can be both adaptive (Davis et al., 1998; Mascaro and Rosen, 2005; King et al., 2006) or maladaptive (Cohen and Cairns, 2012), a series of empirical studies showed that search for meaning in life was overall negatively related to well-being (Steger et al., 2011; Cohen and Cairns, 2012) because people may seek meaning when they are troubled (Thompson and Janigian, 1988). Also, Duffy and Sedlacek (2007) pointed out that search for life meaning reflects a status of less decidedness and comfort in related choices and less clear about their interests and abilities, thus should negatively influence psychological flourishing. Hence, in the current study, we see search for life meaning as a status implying a mismatch between one’s expectation and living status or even a state of confusion about one’s existence. Accordingly, we propose the following sequential mediation hypotheses.

*Hypothesis 3:* Career adaptability and search for meaning in life sequentially mediate the relationships between openness to experience (H3a), conscientiousness (H3b), extraversion (H3c), agreeableness (H3d), neuroticism (H3e), the approach trait (H3f), the avoidance trait (H3g), and psychological flourishing (i.e., individuals’ basic traits → career adaptability → search for meaning in life → psychological flourishing).

## Materials and Methods

### Procedure and Participants

Data were collected from university students in mainland China. We contacted working staff from career centers of several major comprehensive universities in Beijing to email the participation invitation of this study to their undergraduate students. To encourage more participation, we also used snowballing method which means the participants were asked to forward this invitation to their peers. What’s more, to promote students’ active involvement and guarantee questionnaires’ quality, students were informed that the data will be used only for research purpose and we will keep their personal information confidential, moreover, they were rewarded by receiving a debriefing report on their own career adaptability after completing all the questionnaires by email.

To reduce common method bias (Podsakoff et al., 2003), we adopted a time lag of one month between the first wave and the second wave of data collection. At time 1, 194 participants from Chinese universities completed the online questionnaires on demographics, big-five personality, and approach/avoidance traits. After 4 weeks (Time 2), they were reminded by an email to complete the questionnaires on career adaptability, meaning in life, and psychological well-being. As a result, 165 participants (85.1%) provided complete responses, which were used for data analysis. Among the 165 participants, 122 were females (average age = 21.00, *SD* = 1.53) and 43 were males (average age = 21.00, *SD* = 1.47).

### Measures

#### Big-Five Personality

Participants’ big-five personality was measured by the Chinese version of short-form big-five personality scale (Li et al., 2015). This measure showed acceptable internal consistency in openness to experience (α = 0.76), extroversion (α = 0.90), conscientiousness (α = 0.73), neuroticism (α = 0.81), and agreeableness (α = 0.72). Each dimension was measured by three items and participants were asked to rate themselves on a 5-point Likert scale ranging from 1 (*strongly disagree*) to 5 (*strongly agree*).

#### The Approach/Avoidance Traits

Approach/avoidance traits were measured by the 12-item scale developed by Elliot and Thrash (2010), and the Chinese version has been used in previous studies (Guan et al., 2017a). In the current study, participants were asked to rate themselves on a 5-point scale ranging from 1 (*strongly disagree*) to 5 (*strongly agree*) and the Cronbach’s alpha coefficient of six approach items is 0.77 and of the other six avoidance items is 0.87.

#### Career Adaptability

Career adaptability was measured with the Chinese version of the *Career Adapt-Abilities Scale* (Hou et al., 2012), which has demonstrated excellent reliability in previous studies (e.g., Guan et al., 2015c; Yang et al., 2015). It consists of four subscales with six items that measure four dimensions of career adaptability: career concern (e.g., “Thinking about what my future will be like”), career control (e.g., “Keeping upbeat”), career curiosity (e.g., “Exploring my surroundings”), and career confidence (e.g., “Performing tasks efficiently”). Participants responded to each item on a scale from 1 (*not strong*) to 5 (*strongest*). The Cronbach’s alpha coefficient was 0.96 for the total score on all 24 items.

#### Meaning in Life

The MLQ scale was used to measure meaning in life (Steger et al., 2006). This scale contains 10 items, with each of the five items measuring presence of meaning and search for meaning, respectively. Participants were asked to rate on each item from 1 (*absolutely untrue*) to 5 (*absolutely true*). The Cronbach’s alpha coefficient of presence of meaning was 0.81, and the Cronbach’s alpha coefficient of search for meaning was 0.85. A sample item for presence of meaning is “I understand my life’s meaning,” and that for search for meaning is “I am looking for something that makes my life feel meaningful.”

#### Psychological Flourishing

Psychological flourishing was measured with the scale developed by Diener et al. (2010), which contains eight items. Participants were asked to rate on each item from 1 (*strongly disagree*) to 5 (*strongly agree*). The Cronbach’s alpha coefficient was 0.88. A sample item is “My social relationships are supportive and rewarding.”

#### Control Variables

Since previous research has found that age and gender were related to individual well-being (e.g., Shmotkin, 1990; Diener et al., 2002; Burns and Machin, 2010), to rule out the potential confounding effects, we incorporated these variables as controlling variables in our model: age and gender (0 = male, 1 = female).

## Results

### Descriptive Statistics and Correlations

The descriptive statistics and correlations among variables are showed in **Table 1**.

**Table 1:** Descriptive statistics, reliability coefficients, and inter-correlations among variables.

	*Mean*	*SD*	1	2	3	4	5	6	7	8	9	10	11	12	13
1. Age	20.64	1.51	**NA**												
2. Gender	1.74	0.44	0.02	**NA**											
3. Openness	3.58	0.71	0.06	-0.08	**0.76**										
4. Extraversion	3.09	0.94	-0.03	0.04	0.35**	**0.90**									
5. Agreeableness	3.80	0.58	0.01	-0.03	0.03	0.23**	**0.72**								
6. Consciousness	3.45	0.63	0.09	0.10	0.09	0.18*	0.32**	**0.73**							
7. Neuroticism	3.10	0.77	0.05	0.01	-0.36**	-0.51**	-0.19*	-0.18*	**0.81**						
8. Approach trait	3.89	0.50	-0.03	0.15	0.34**	0.26**	0.10	0.21**	-0.14	**0.77**					
9. Avoidance trait	3.02	0.71	-0.03	-0.03	-0.22**	-0.29**	-0.10	-0.21**	0.64**	-0.04	**0.87**				
10. CA	3.70	0.53	0.14	-0.03	0.38**	0.28**	0.22**	0.40**	-0.39**	0.43**	-0.43**	**0.96**			
11. PMIL	3.51	0.66	0.15*	0.03	0.30**	0.25**	0.16*	0.20*	-0.34**	0.43**	-0.36**	0.65**	**0.81**		
12. SMIL	3.65	0.67	0.00	0.02	0.11	0.01	0.01	0.07	-0.14	0.26**	-0.07	0.31**	0.26**	**0.85**	
13. PF	3.72	0.60	0.03	0.01	0.27**	0.48**	0.33**	0.34**	-0.45**	0.37**	-0.36**	0.66**	0.57**	0.23**	**0.88**

*^∗^p < 0.05, ^∗∗^p < 0.01, ^∗∗∗^p < 0.001, N = 165. Reliability coefficients are shown in bold along the diagonal of the table. CA, career adaptability; PMIL, presence of meaning in life; SMIL, search for meaning in life; PF, psychological flourishing.*

### Examining the Sequential Mediation Model

To examine the sequential mediation hypotheses, we adopted the procedure proposed by Preacher and Hayes (2008). Accordingly, three criteria should be justified: firstly, the independent variables (big-five personality, approach trait and avoidance trait) should significantly correlate with mediator variable (career adaptability and meaning in life); secondly, after controlling the effect of the independent variable, the correlation between mediator variable and dependent variable (psychological flourishing) should be significant; thirdly, the indirect effect from independent variable to dependent variable should be significant. PROCESS procedures were used to examine the significance of indirect effects. Before analyses, all continuous predictors including control variables were centered (Aiken and West, 1991).

**Table 2** presents the results of hierarchical regression analyses. After controlling the effects of age and gender, career adaptability is significantly predicted by openness to experience (β = 0.12, *p* < 0.05), consciousness (β = 0.21, *p* < 0.001), the approach trait (β = 0.38, *p* < 0.001), and the avoidance trait (β = -0.24, *p* < 0.001). And, career adaptability positively predicts presence of meaning in life (β = 0.57, *p* < 0.001) and search for meaning in life (β = 0.35, *p* < 0.01), after controlling for the effects of independent variables, age, and gender, yielding support for hypotheses 1a, 1b, 1e, 1g, but not 1c, 1d, and 1f, as the effects of extraversion, neuroticism, and agreeableness on career adaptability are not significant in this model. Furthermore, after controlling the effects of age, gender, basic traits, and career adaptability, presence of life meaning showed significant correlation with psychological flourishing (β = 0.18, *p* < 0.01), while no significant effect was found for search for life meaning (see **Table 2**).

**Table 2:** Examining the sequential mediation model.

Variables	Career adaptability	Presence of meaning in life	Search for meaning in life	Psychological flourishing
Constant	0.95	0.04	2.64	0.98
Age	0.05	0.04	-0.01	-0.02
Gender	-0.13	0.03	0.02	-0.01
Openness	0.12*	-0.01	-0.04	-0.06
Extraversion	-0.01	0.00	-0.10	0.16
Agreeableness	0.07	0.05	-0.05	0.12
Consciousness	0.21***	-0.12	-0.06	0.05
Neuroticism	-0.05	-0.06	-0.16	-0.08
Approach trait	0.38***	0.30**	0.23	0.04
Avoidance trait	-0.24***	-0.10	0.11	0.02
CA		0.57***	0.35**	0.39***
PMIL				0.18**
SMIL				0.04
*R*^2^	0.46	0.48	0.15	0.58
Adjust *R*^2^	0.43	0.45	0.09	0.55
*F*	18.20***	41.46***	9.43***	4.18*

*^∗^p < 0.05, ^∗∗^p < 0.01, ^∗∗∗^p < 0.001, N = 165.*

**FIGURE 2:**
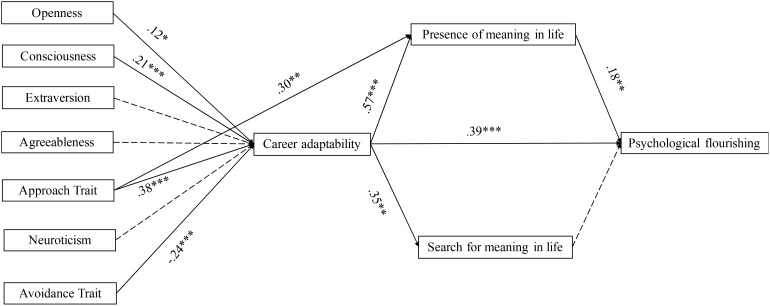
The sequential mediation model. ^∗^*p* < 0.05, ^∗∗^*p* < 0.01, ^∗∗∗^*p* < 0.001; *N* = 165. Paths in dotted lines are not significant.

Indirect effects were calculated with bootstrapping analyses in PROCESS (see **Figure 2**). Specifically, the indirect effects of openness to experience [95% CI = (0.00, 0.04)], consciousness [95% CI = (0.01, 0.05)], the approach trait [95% CI = (0.01, 0.09)], and the avoidance trait [95% CI = (-0.06, -0.01)] on psychological flourishing through career adaptability then presence of meaning were significant, which supports hypotheses 2a, 2b, 2e, and 2g, but not 2c, 2d, and 2f. What’s more, since there are no main effects of search for meaning on well-being, all the mediations (Hypothesis 3) through it were not significant (see **Figure 2**).

## Discussion

Based on career construction theory, the current study examined a sequential mediation model for the relations among indicators of adaptivity (big-five personality and approach/avoidance traits), career adaptability, meaning in life, and individual well-being among Chinese university students. The two-wave survey study showed that career adaptability and presence of life meaning mediated the effects of openness to experience, consciousness, approach trait, and avoidance trait on individual well-being in sequence. Theoretical and practical implications of these findings are discussed below.

First, career construction theory (Savickas, 2005, 2013) maps the pathway from career adaptivity to adaptability, adaptive behaviors, and adaptation results; though previous research has identified that career adaptability is significantly related to well-being, not much work has been done to test the proposition that career adaptability serves as the key explanatory link that mediates the effects of career adaptivity indicators on well-being (Hartung and Taber, 2008). The current study helps to address this question by showing how career adaptivity indirectly affects well-being through career adaptability, which also offers an important career construction perspective on the mechanisms underpinning university students’ well-being. In line with career construction perspective, future studies could extend this line of research by incorporating other important personal characteristics, such as proactive personality.

Second, while previous studies have established the impacts of career adaptability on individual well-being (Rudolph et al., 2017), it is still unknown why this is the case. Hence, we contribute to the career construction literature by explicating presence of life meaning as one mechanism, through which career adaptability leads to a sense of well-being. These findings not only repeatedly verified the core stipulation of career construction theory (Savickas, 2005, 2013), but also provided an insightful approach to understand how career adaptability ultimately impact individuals’ well-being.

Third, our research demonstrated that career adaptability and presence of meaning in life mediated the effects of career adaptivity on psychological flourishing in sequence. These findings suggest that career construction theory can serve as an important theoretical framework to guide the investigation on the antecedents of individual well-being (Hartung and Taber, 2008). In addition, the findings also contribute to well-being research by considering psychological flourishing as the indicator, which responded to the call to use integrative indicator to enhance the accuracy and comprehensiveness in well-being studies (Huppert and So, 2013).

What’s more, as shown in **Figure 2**, the current study showed that approach trait’s effect on presence of meaning was not fully mediated by career adaptability. It is plausible that approach trait may create an influx of positive emotions (e.g., optimism), which ultimately leads to gains in life meaning (Lavigne et al., 2013). Besides, in our study, career adaptability’s effect on psychological flourishing was not fully mediated by presence of meaning. This may be due to the fact that higher levels of career adaptability showed also higher positive psychological characteristic such as hope, which is helpful to promote psychological flourishing (Diener et al., 2010).

However, in the present study, the effects of extraversion, neuroticism, and agreeableness on career adaptability were not significant. A plausible explanation may be that the other four individual characteristics included in this study were relatively more salient predictors than extraversion, neuroticism, and agreeableness. The fact that extraversion, neuroticism, and agreeableness significantly predicted career adaptability on a bivariate level speaks to these potential explanations. In addition, consistent with prior research (Teixeira et al., 2012; Li et al., 2015), the effects of agreeableness on career adaptability were found to be non-significant, which may also reflect that agreeableness has mixed effects on career adaptability (Guan et al., 2017a). In this case, it is possible that its effects on career adaptability are not straightforward, so we encourage future research to further explore possible mechanisms that can address this issue.

We also did not find support for Hypothesis 3, which proposed that search for life meaning would negatively mediate the relationships between career adaptability and well-being. A possible explanation may be that the effect of search for life meaning is contingent on other variables (Steger et al., 2008), such as personal characteristics, e.g., need for cognitive closure, which captures the desire to reduce uncertainty and ambiguity and to reach closure on judgments and decisions (Kruglanski, 2004). Accordingly, people search for meaning when they are unsure about meaning in their lives. For students with a higher need for cognitive closure, who have a low tolerance for uncertainty, they could presumably experience lower levels of well-being because of frustrated cognitive needs when searching for life meaning. While students with a lower need for cognitive closure are prone or able to flexibly change their perceptions, thus being more receptive and responsive to uncertainties during the searching process. In this case, their sense of well-being may be less influenced. Future research could explore other possible moderators of the relationship between search for life meaning and individual well-being.

The current research also carries important practical implications for career education and career consulting. As our results showed, openness to experience, conscientiousness, and approach/avoidance traits can significantly predict career adaptability, meaning in life and individual well-being. Educators thus can use these traits to diagnose and identify students who have risk of poor career adaptability and low well-being in the university. Furthermore, for students with low levels of well-being, it’s important to strengthen, in particular, their career adaptability. Career counselors could implement relevant inventions, for example, by helping students observe role models, making more appropriate self-assessment, and career planning to cultivate their career adaptability and stimulate their self-fulfillment. Through these practices, career educators, and counselors could identify university students’ career adaptability and sense of life meaning, thus helping them enjoy university life and better achieve life effectiveness.

Despite the theoretical and practical implications discussed above, the current study has some limitations. First, snowballing sampling method was used to collect the data and the participants didn’t report their universities and majors, which may influence the results’ generalizability. Because uneven development of regional economy and education background may lead students to view well-being in different ways. Future research may need to focus on a more representative sample in order to make the results more convincing. Besides, this study was conducted among a sample of Chinese university students, who are at the beginning of their career and have less financial strain, work pressure, or other employment problems. Thus, whether the current results can be generalized to samples from other countries remains to be examined in future research. For example, Chinese culture is characterized by high levels of collectivism, power distance, and long-term orientation, and it has been found that Chinese university students’ career decisions are more likely to be influenced by social contexts than American students (Guan et al., 2015a). It is possible that the roles of individual traits on their career adaptability and well-being are more influenced by social environments, such as parental influence and peer influence, in the Chinese context (Guan et al., 2015b). Future research should continue to examine how the current model is influenced by cultural environments. Second, although we adopted a two-wave research design to reduce common method bias (Podsakoff et al., 2003), this design cannot draw causal conclusions from current results. To address question of causality, experimental, or longitudinal designs should be used in future research. Third, self-report measures were used in this study, which may risk the validity of the findings. However, since all the variables pertain to individual perceptions about their own status, we believe self-report can be the appropriate method. In future studies, the inclusion of social desirability as control variable can be considered.

## Ethics Statement

This study was carried out in accordance with the recommendations of ethics committee of Peking University with written informed consent from all subjects in accordance with the Declaration of Helsinki. The protocol was approved by the ethics committee of Peking University.

## Author Contributions

MZ and ZS led the literature review, research design, data analysis, and paper drafting work for this paper. ZC and ZH made contributions in data analysis and paper revision. QX, PW, and FZ made contributions in data collection and paper drafting.

## Conflict of Interest Statement

The authors declare that the research was conducted in the absence of any commercial or financial relationships that could be construed as a potential conflict of interest. The reviewer ZW and handling Editor declared their shared affiliation.
